# Supplementary material for: Automated phenotyping of postoperative delirium-like behaviour in mice reveals the therapeutic efficacy of dexmedetomidine
Source: Commun Biol. 2023 Aug 2;6:807. doi: 10.1038/s42003-023-05149-7 (PMC10397202; doi:10.1038/s42003-023-05149-7)
Supplement: Supplementary file 2 — Supplementary Information [file 42003_2023_5149_MOESM2_ESM.pdf]

Supplement Figure and legends

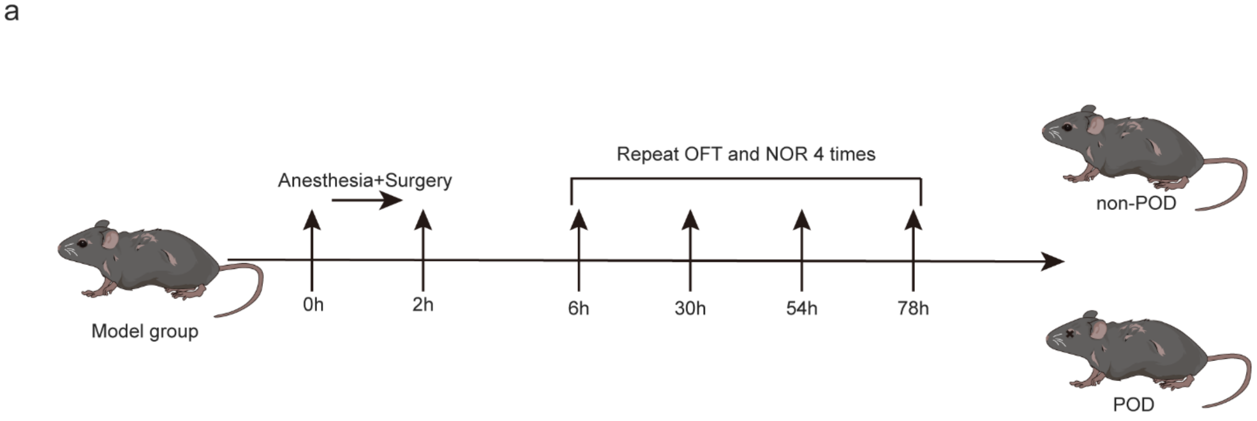

Figure S1. Flowchart of model composition methodology.

(a) Schematic diagram of the experimental design timeline.

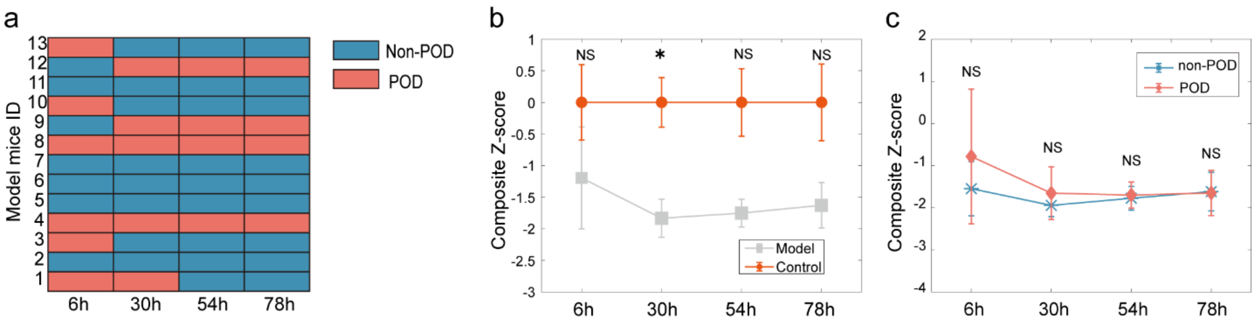

Figure S2. Evaluation of classical index results. a) The incidence of different mice at different time points at the individual level. The matrix pixels represent the incidence of 52 pairs of mice according to the clustering. Results in dark blue means the mice at this time is falling in POD; the light colour means the mice at this time is falling in non-POD. The non-jumping display results in the matrix show the reliability of the clustering results. **b)** Summary of composite Z_{score} in the control and model groups. One time point showed a significant difference between the two groups (30 h, control=0±0.392, model=-1.833±0.302). Statistics: two-way ANOVA followed by the

Sidak post hoc multiple comparison test, *30 h, P=0.0486. **c)** Summary of composite Z_{score} in non-POD and POD mice. The bold traces and shadows indicate the mean ± SEM. The results show no significant differences between the two groups. Statistics: two-way ANOVA followed by the Sidak post hoc multiple comparison test.

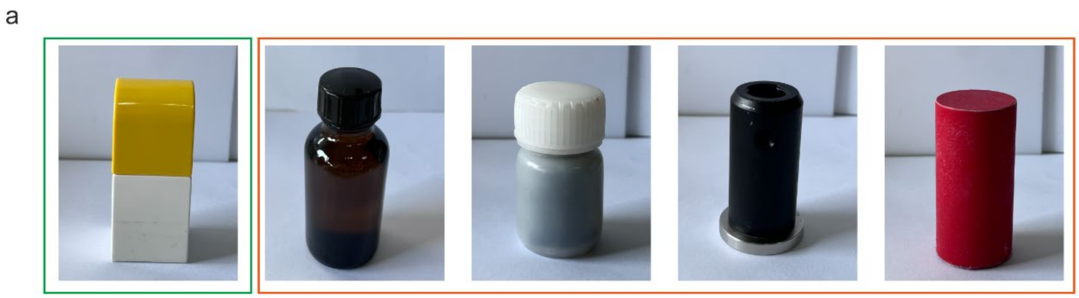

Figure S3. Evaluation of for the task-driven behaviour. a) Five of the objects used in the NOR tests. The object shown in the green square is a familiar object and those shown in the orange rectangle are novel objects. They are used, from left to right, for 6 hours, 30 hours, 54 hours, 78 hours. In case we needed the novel objects, what we need is to ensure that the new objects are similar in shape, with different materials and colours.

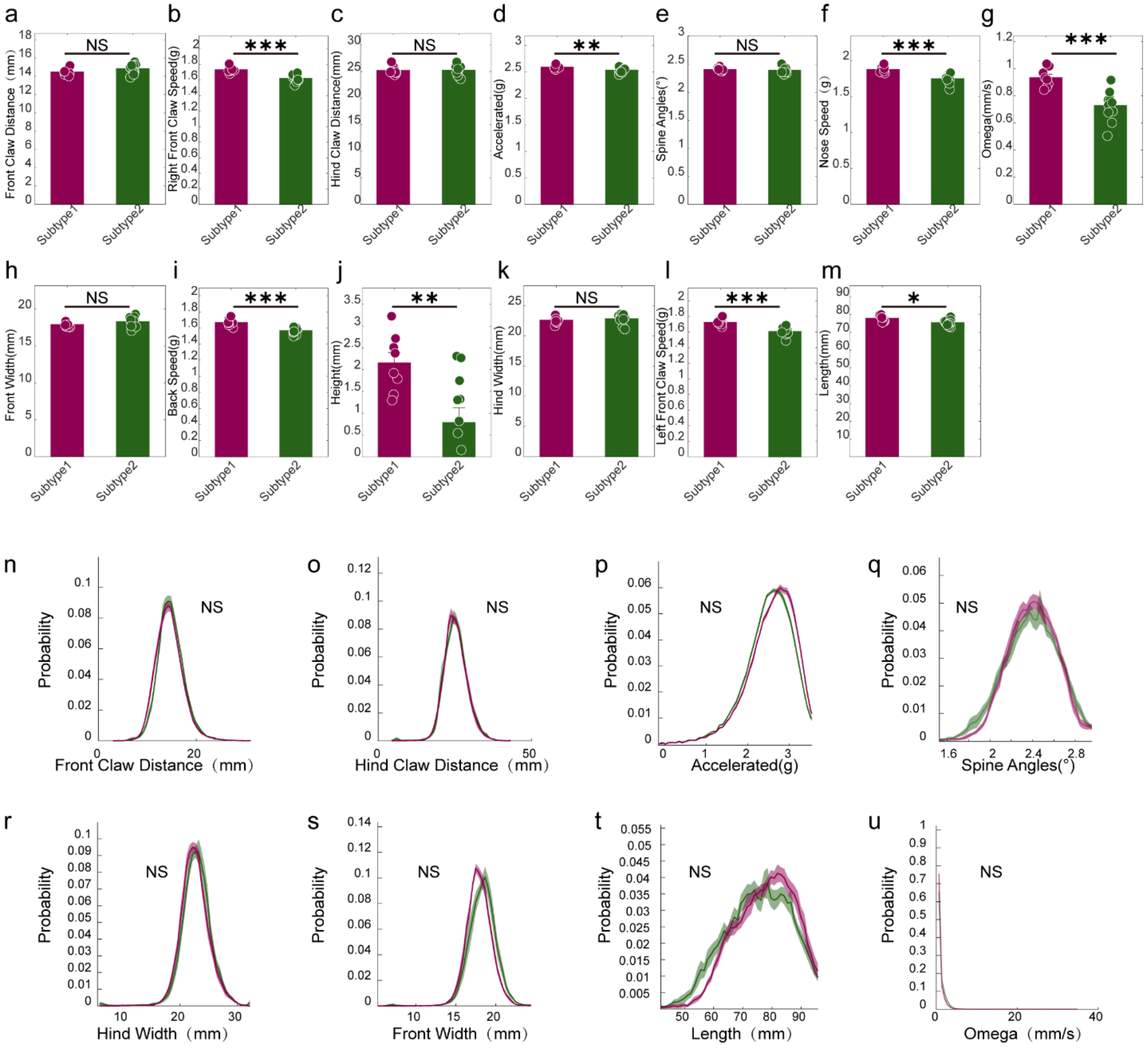

Figure S4. Evaluation of for the pose parameters. a-m) Box plots of the front claw distance (NS, *P*=0.095), hind claw distance (NS, *P*=0.958), accelerated (***P*=0.004), spine angle (NS, *P*=0.436), front width (NS, *P*=0.106), hind width (NS, *p*=0.453), length (***P*=0.005), left front claw speed (*****P*<0.0001), back speed (*****P*<0.0001), height (***P*=0.007), nose speed (*****P*<0.0001), omega (*****P*<0.0001), and right front claw speed (*****P*<0.0001) of the two groups of animals (purple: hyper, *n*=11; green, hypo, *n*=11; statistics: two-sided unpaired *t*-test), values are represented as mean ± SEM. **n-u)** Comparisons of PMF of certain kinematic or posture parameters

for cluster1 and cluster2, including the front claw distance (*P*=0.095), hind claw distance (*P*=0.095), accelerated (*P*=0.999), spine angle (*P*=0.560), front width (*P*=0.612), hind width (*P*=0.172), length (*P*=0.069), and omega (*P*=0.129). The Kolmogorov-Smirnov test was used to test for differences between the distributions of the two clusters. The bold traces and shadows indicate the mean ± SEM. The fractions of each group and light colour traces are the fractions of all 19 mice (purple, hyper, *n*=11; green, hypo, *n*=8).

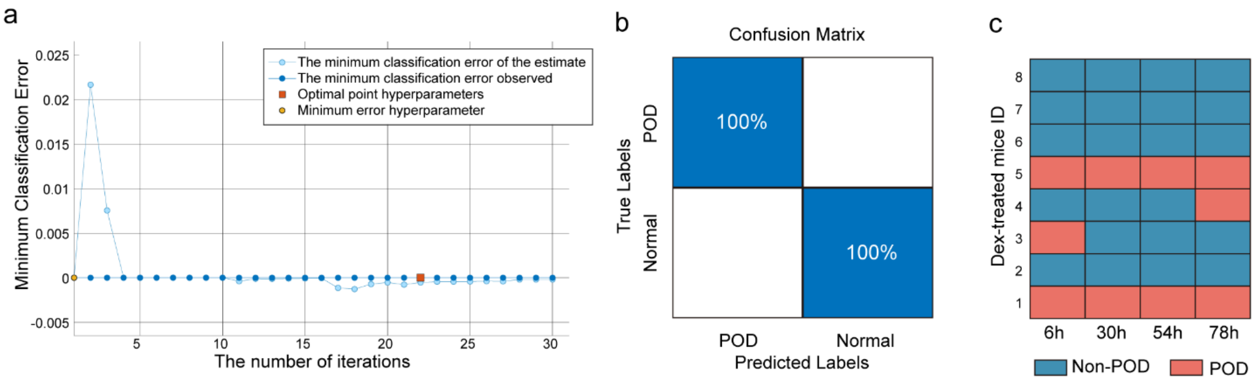

Figure S5. Behavioural evaluation and machine learning-based model evaluation. a) Minimum classification error plot. **b)** Confusion matrix plot. **c)** The incidence of different mice at different time points at the individual level. The matrix pixels represent the incidence of 32 pairs of mice, according to the clustering results. The dark colour means the mice at this time is falling in POD; the light colour means the mice at this time is falling in non-POD. The non-jumping display results in the matrix show the reliability of the clustering results.
