# Supplementary material for: Automated phenotyping of postoperative delirium-like behaviour in mice reveals the therapeutic efficacy of dexmedetomidine
Source: Commun Biol. 2023 Aug 2;6:807. doi: 10.1038/s42003-023-05149-7 (PMC10397202; doi:10.1038/s42003-023-05149-7)
Supplement: Supplementary file 3 — Description of Additional Supplementary Files [file 42003_2023_5149_MOESM3_ESM.pdf]

Description of Additional Supplementary Files

File name: Supplementary Data 1
Description: Source data used to generate bar figures.
